# Neurorehabilitation and Music

**Published:** 2016

**Authors:** VL Purcarea

**Affiliations:** * “Carol Davila” University of Medicine and Pharmacy, Bucharest, Romania

The exciting end of the year was, for the medical world, full of moments of reunion and also moments of professional and human dedication.

The 3rd European Congress of NeuroRehabilitation, which took place between 1st and 4th of December 2015, in Hofburg Center, Vienna, Austria, should be mentioned among the many professional events which took place last year. The event was organized by the European Federation of NeuroRehabilitation Societies in collaboration with the Austrian Society of NeuroRehabilitation and the German Society of NeuroRehabilitation.

Moreover, the event was envisioned and turned out to be a binder for the remarkable specialists in the field and had the following main theme “Transition and Translation: Two challenges for NeuroRehabilitation”.

The latest accomplishments in neurorehabilitation research were presented during the event. The participation of a great number of renowned speakers raised to excellence the scientific quality of the congress manifestations.

**Fig. 1:**
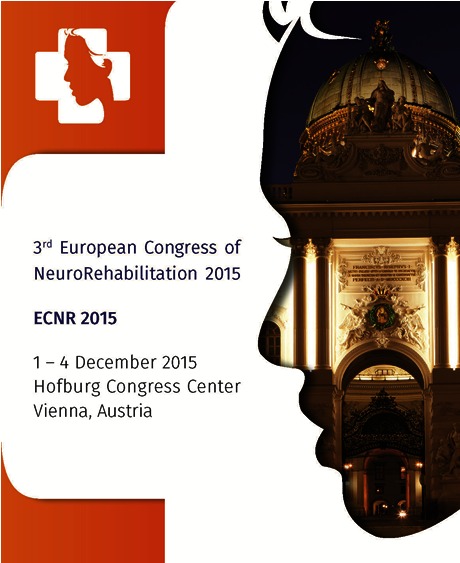
**Fig. 1**3rd European Congress of NeuroRehabilitation, Vienna 2015

Among the most important plenary lectures of the 3rd European Congress of NeuroRehabilitation the following should be mentioned: “Advances in neurorehabilitation fundamentals – an update”, Dafin Muresanu, Cluj Napoca, Romania; “Investigating anhedonia with whole-brain computational connectomics: Avenues for a new psychiatry?”, Leopold Saltuari, Austria; “The neurophysiology of beauty”, Semir Zeki, UK; Heinrich Binder, Austria; “Memory disorders and the concept of reality” – “Psychogenic amnesia”, Michael Kopelman, UK; “Determinants of reality distortion in psychosis”, Werner K. Strik, Czech Republic; „Neurorehabilitation in spinal cord injury” – „An update on perspectives: stem cells, nogo and so on”, Armin Curt, Switzerland; „Neuromodulation by manipulating proprioception” – “Neurochemistry and connectivity of the propriospinal system”, Peter Riederer, Germany; “Focal vibration in neurorehabilitation”, Frederic Albert, France; “Advances in neuroprosthetics and mind-brain interfaces” – “Bionic Eye”, Susanne Binder, Austria; “Cochlea-Implants”, Thomas Lenarz, Germany; “Biokinetic rehabilitation after stroke”, chairs Mario Siebler, Germany and Hermann Moser, Austria; “Parkinson disease: new advances in treatment and rehabilitation”, chairs Wassilios Meissner, France and Giovanni Abbruzzese, Italy. 

During the event, the following “Meet the Experts Sessions” took place: “Pharmacology in neurorehabilitation”, Volker Homberg, Germany; “Early neurorehabilitation: do we need specialization at ICU or at rehabilitation unit?, Heinrich Binder, Austria, etc., and also “The Free Communication Sessions” among which the following should be mentioned: “Neuromodulation, pharmacology and post-surgical rehabilitation” – “Neuromodulation with whole-hand electrical stimulation in stroke patients in subacute stage”, K. Schwenker, M. Christova, H. Bartsch, M. Seidl, L. Koenig, A. Kunz, E. Trinka, F. Gerstenbrand, S. Golaszewski, Austria, etc.

Moreover, issues of public health were also approached: “Analysis of the outcome in home healthcare services in Abruzzo region (Italy): survey on district”, R. Saggini, R.G. Bellomo, E. Ancona, S.M. Carmignano, A. Di Stefano, G. Barassi, Italy.

Also, the ENFR Awards for Young Researchers endowed with EUR 500 – each, were awarded to the 2 best abstracts and were presented during the Closing Session on the 4th of December 2015.

**Fig. 2:**
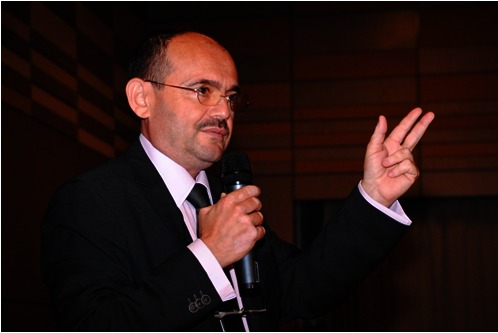
**Fig. 2**Prof. Dafin Fior Muresanu, MD, the newly elected Vice-president of the European Federation of NeuroRehabilitation Societies

As a confirmation of his scientific recognition and international prestige, on this occasion, the famous Prof. Dafin Fior Muresanu, MD, President of the Romanian Society of Neurology was elected Vice-president of the European Federation of NeuroRehabilitation Societies.

What should also be mentioned as belonging to the moments of human dedication in the diversity of the medical world is the “Christmas and New Year’s Festive Concert” of “Dr. Ermil Nichifor” Physicians Orchestra in Bucharest under the inspired baton of the orchestra conductor, Iosif Ion Prunner, on the 27th of December, in Romanian sanctuary of classical music, the Romanian Athenaeum. 

**Fig. 3:**
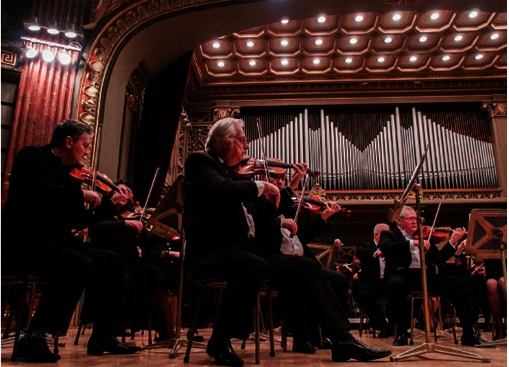
**Fig. 2**“Dr. Ermil Nichifor” Physicians Orchestra in Bucharest

**Fig. 4:**
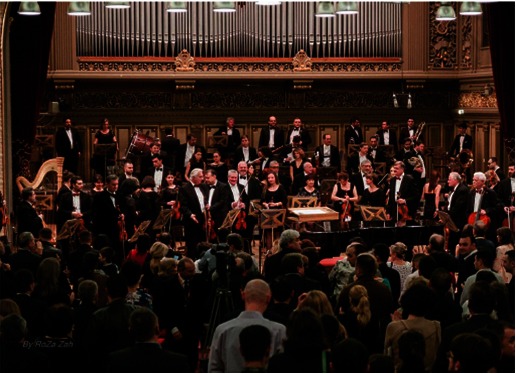
**Fig. 2**Iosif Ion Prunner, orchestra conductor of “Dr. Ermil Nichifor” Physicians Orchestra

The artistic program also included some important music pieces belonging to the universal classic music treasure, such as: J. STRAUSS (Viennese Blood and Blue Danube waltzes, Hunting Polka and Thunder and Lightning Polka), G. VERDI (La Traviata, Prelude to Act 1), P.I. CEAIKOVSKI (Sleeping Beauty Waltz), P. MASCAGNI (Intermezzo from Cavalleria Rusticana), SOSTAKOVICI (Waltz 2), E. DOGA (The waltz of the century), L. ANDERSON (A Christmas Festival), all being interpreted devotedly and with talent and profusely offered to a connoisseur auditorium. 

**Fig. 5:**
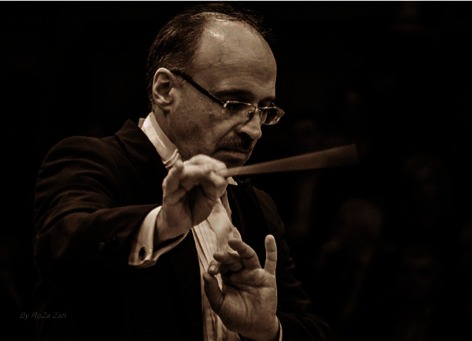
**Fig. 5**“Dr. Ermil Nichifor” Physicians Orchestra in Bucharest, “Christmas and New Year’s Festive Concert” at the Romanian Athenaeum

The Concert’s exceptional success was also guaranteed by the remarkable contribution of the opera singers such as Alina Bottez (Santuzza from Cavalleria Rusticana), Irina Iordachescu (Ave Maria from Otello), Oana Andra (Musette Waltz from La Boheme), Catalin Toropoc (Felice ancor io son? Per me e giunta il di supremo Aria from Don Carlo) and Adrian Dumitru (Calaf Aria – Nessun dorma from Turandot).

**Fig. 6:**
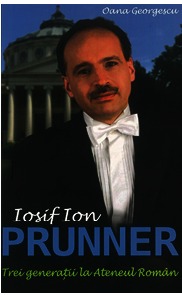
**Fig. 6**Book “Iosif Ion PRUNNER – Three generations at the Romanian Athenaeum”

At the end of the wonderful Concert, the book “Iosif Ion PRUNNER – Three generations at the Romanian Athenaeum” was launched in front of an impressive public. 

**Fig. 7:**
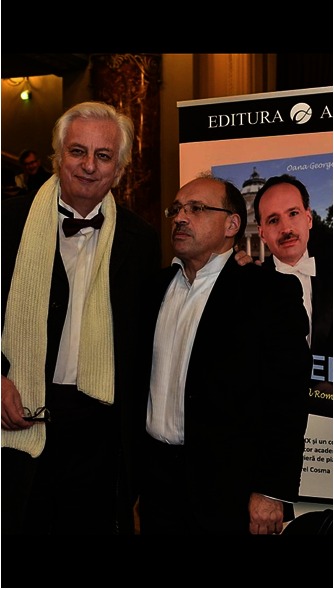
**Fig. 7**Head of “Dr. Ermil Nichifor” Physicians Orchestra, Prof. Mircea Penescu, MD and orchestra conductor Iosif Ion Prunner

Executive Editor

 Prof. Dr. Eng. Victor Lorin Purcarea

**Executive Editor** **Assoc. Prof. Dr. Eng. Victor Lorin Purcarea**

